# In Which Patients with Sarcoidosis Is FDG PET/CT Indicated?

**DOI:** 10.3390/jcm9030890

**Published:** 2020-03-24

**Authors:** Ruth G.M. Keijsers, Jan C. Grutters

**Affiliations:** 1 Department of Nuclear Medicine, St Antonius Hospital, Koekoekslaan 1, 3435 CM Nieuwegein, The Netherlands; 2 Interstitial Lung Diseases Center of Excellence, Department of Pulmonology, St Antonius Hospital, Koekoekslaan 1, 3435 CM Nieuwegein, The Netherlands; j.grutters@antoniusziekenhuis.nl; 3 Division of Heart & Lungs, University Medical Center Utrecht, Heidelberglaan 100, 3584 CX Utrecht, The Netherlands

**Keywords:** sarcoidosis, FDG PET/CT

## Abstract

Sarcoidosis is a granulomatous disease of which the etiology remains unknown. The diverse clinical manifestations may challenge clinicians, particularly when conventional markers are inconclusive. From various studies, it has become clear that fluorodeoxyglucose (FDG) positron emission tomography (PET)/CT aids in sarcoidosis care. In this article, an update on FDG PET/CT in sarcoidosis is provided. The use of FDG PET/CT in the diagnostic process of sarcoidosis is explained, especially in determining treatable inflammatory lesions in symptomatic patients with indecisive conventional tests. Furthermore, FDG PET/CT for evaluating the potential benefit of additional inflammatory treatment is described and its use in cardiac sarcoidosis is highlighted.

## 1. Introduction

Sarcoidosis is a granulomatous disease of unknown etiology with heterogeneous clinical manifestations. Different organs may be affected, and the intensity of inflammation often varies throughout the body. The natural evolution of the disease and its response to treatment is highly variable. In addition, the extent of tissue damage and fibrosis as a result of the inflammatory process differs between patients, even within patients per affected organ or tissue. There may be symptoms related to sarcoidosis but not necessarily to the presence of granulomatous inflammation or the deposition of granulomata in a specific organ, such as chronic fatigue, small fiber neuropathy, and vitiligo. The wide spectrum of symptoms, functional sequelae, and related loss of wellbeing for patients challenges health care professionals in diagnosing and assessing disease activity in everyday practice. Clinical features (e.g., erythema nodosum, uveitis, or changing scar due to localization of granulomas), biochemical markers (e.g., elevated serum angiotensin converting enzyme (ACE), serum or urine calcium, or lymphocytes in the bronchoalveolar lavage fluid), and abnormalities/changes in imaging (chest radiography, CT, or MRI) are conventionally used to assess (change of) disease activity. However, these markers have a limited diagnostic performance.

Fluorodeoxyglucose (FDG), labeled with the positron emitting fluorine-18 (18F), is a synthesized glucose analogue and well known for its application in a wide variety of clinical conditions, such as cancer. FDG positron emission tomography (PET) studies show a high signal-to-noise ratio and deliver high contrast images of metabolic active granulomatous disease. From various studies over the last decade and from our clinical experience, it is thought that FDG PET can be helpful in sarcoidosis management, especially in the assessment of organ-specific disease activity [1]. In this article, the current role of FDG PET/CT in the diagnosis and management of sarcoidosis is explained. Precision treatment based on FDG PET/CT is discussed (i.e., through localizing sites of active inflammation), and its role in identifying patients that may respond to anti-inflammatory therapy is highlighted.

## 2. Initial Studies of FDG PET/CT in Sarcoidosis

In sarcoidosis, the granuloma includes CD4+ lymphocytes and activated macrophages. Like malignant cells, these leukocytes express glucose transporters (GLUTs) in the cell membrane, particularly GLUT-1 and GLUT-3 [2]. Analogous to glucose, FDG is transported into lymphocytes and macrophages through these GLUTs. Therefore, FDG PET can be used in leukocyte-mediated diseases, like sarcoidosis and other granulomatous disorders [3]. The use of FDG PET in sarcoidosis was first reported by Lewis and Salama [4], and since then, several clinical studies have illustrated that FDG uptake in sarcoidosis represents active granulomatous inflammation.

Teirstein was the first to report the results of whole-body FDG PET scans in a large cohort of 137 sarcoidosis patients. Positive pulmonary FDG PET findings occurred in two-thirds of patients with radiographic stage II and III sarcoidosis. Negative pulmonary FDG PET findings were common in patients with radiographic stage 0, I, and IV sarcoidosis [5].

Disease duration might influence these results of FDG PET/CT, since sarcoidosis in remission as well as end-stage fibrosis may show anatomical abnormalities while the disease itself has become inactive. Therefore, several studies evaluated the sensitivity of FDG PET in newly diagnosed and histologically proven sarcoidosis, considering this as the gold standard for active disease. FDG PET and the next generation FDG PET/CT scanners were evaluated not only in pulmonary sarcoidosis but in extra pulmonary disease as well.

Table 1 presents an overview of these studies with a sensitivity of FDG PET or FDG PET/CT for active sarcoidosis ranging between 89% and 100% [5,6,7,8,9,10,11,12,13,14,15,16,17].

## 3. FDG PET/CT Findings and Histological Confirmation of Sarcoidosis

The studies presented in Table 1 used the histological confirmation of sarcoidosis as a gold standard in determining the sensitivity of FDG PET/CT in sarcoidosis. Subsequently, the use of FDG PET/CT in guiding clinicians for biopsy sites in order to obtain histological proof of sarcoidosis was evaluated. FDG PET/CT guided mediastinoscopy as well as video-assisted thoracoscopic surgery (VATS) demonstrated that metabolic active lesions represented non-caseating granulomas matching sarcoidosis [7,18].

In clinically relevant organ involvement like cardiac or neurosarcoidosis, histological proof of sarcoidosis is highly preferred. The presence of a typical sarcoid uptake pattern on whole body FDG PET/CT—like active mediastinal and bilateral hilar lymph nodes, whether or not combined with lung parenchymal active disease—supports the likelihood of sarcoidosis. Instead of a myocardial or cerebral biopsy, FDG PET/CT may reveal more easily accessible but clinically concealed organ or tissue involvement. Simonen et al. evaluated 68 patients with cardiac sarcoidosis [7]. To assess the presence of active systemic disease, 24 patients with metabolically active thoracic lymphadenopathy imaged by FDG PET underwent mediastinoscopy. In all patients, the presence of non-caseating granulomas was confirmed.

This approach was also successful in cases initially presenting with symptoms suggestive of neurosarcoidosis [19,20] and chronic uveitis [21,22]. In a study by Rahmi et al., 17 of the 54 consecutive patients with chronic uveitis (31.5%) presented with hypermetabolic foci on FDG PET consistent with sarcoidosis [23]. Similar findings were observed in 10 of 19 patients (53%) with unexplained chronic uveitis and normal chest CT [24].

## 4. FDG PET/CT Compared to Conventional Markers of Sarcoidosis Activity

In the past decade, several serum markers for disease activity have been investigated in sarcoidosis. These markers can be divided into three groups: macrophage and granuloma associated, lymphocyte associated, and extracellular matrix associated [25].

ACE is a macrophage/granuloma associated marker. It can be produced by epithelioid cells and activated macrophages, and increased serum levels are thought to represent the total burden of granuloma [26]. Although ACE is probably the most widely used serological test in sarcoidosis, its diagnostic value is limited. On average, the sensitivity of the test to detect activity lays around 50%. Of note, the application of ACE insertion/deletion genotype correction may improve the test performance significantly [27,28,29,30,31,32]. Although elevated ACE cannot predict disease outcome, repeated measurement can nevertheless be useful for treatment monitoring [33,34].

Soluble interleukin-2 receptor (sIL-2R) is a lymphocyte associated marker. IL-2 receptors are found on the surface of T lymphocytes, B lymphocytes, monocytes, and macrophages [35,36,37]. sIL-2R’s soluble form is associated with the activation of the cellular immune system and therefore is probably increased in sarcoidosis. The sensitivity of the serum sIL-2R test to detect disease activity is approximately 70% and appears higher in patients with lung involvement [38,39]. However, sIL-2R does not correlate with disease severity, and conflicting results have been found regarding the correlation between sIL-2R and long-term outcome, for example, of lung function [38,40].

Several studies have correlated ACE and sIL-2R test results with FDG PET. In 36 patients with recent onset sarcoidosis, 34 (94%) showed metabolic active disease on FDG PET. ACE and sIL-2R were increased in only 36% and 47% of the patients, respectively [14].

Mostard et al. analyzed 89 chronic sarcoidosis patients with persisting disabling symptoms. Active disease imaged by FDG PET/CT was found in 65 patients (73%) [41]. In this group, only 22% showed an elevated ACE level, while sIL-2R was increased in 68%. Remarkably, none of the 24 patients without metabolic activity on FDG PET/CT had an increased ACE or sIL-2R. Sobic-Saranovic et al. found similar discrepancies between ACE and FDG PET/CT. In their group with positive FDG PET/CT lesions, only 37 patients (49%) had increased ACE levels [42].

Comparison of chest radiography with FDG PET in sarcoidosis has illustrated the difference between anatomical and functional imaging. Staging of pulmonary disease based on chest X-ray did not correlate well with active disease locations imaged by FDG PET [15,41,43]. Based on FDG PET, the majority of patients with radiographic stage 0 were in fact stage I, while stage I frequently appeared to be stage II, representing parenchymal involvement. In addition, patients with radiographic stage IV disease (i.e., signs of fibrosis) regularly showed active parenchymal disease. The latter finding is relevant since the continuous inflammation might lead to additional destruction of the lung.

The aforementioned studies suggest FDG PET/CT to be superior in evaluating disease activity compared to ACE, sIL-2R, and chest radiography.

Besides its value as a marker of sarcoidosis activity, FDG PET/CT has been shown to contribute to solving complex clinical cases. Braun et al. provided relevant data on the diagnostic value of FDG PET/CT in a series of 20 patients, particularly in atypical, complex, and multisystemic forms of sarcoidosis, like sinonasal, pharynx/larynx, stomach, liver as well as lacrimal and salivary gland involvement [13]. In patients with known sarcoidosis and newly onset spinal symptoms, FDG PET was able to detect active spinal sarcoidosis and correlated with the clinical response to immunosuppressive treatment [44]. Additionally, the diagnostic value of FDG PET/CT has been demonstrated in bone and bone marrow involvement [45,46], sarcoidosis-associated fasciitis [47], giant cell vasculitis [48,49,50], sarcoid myositis [51,52], epididymal involvement [53], and laryngeal sarcoidosis [54]. In cases with fever of unknown origin or unexplained 1,25-dihydroxyvitamin D-mediated hypercalcemia, increased renal or splenic FDG uptake was present and led to the diagnosis of sarcoidosis [55,56].

Anemia may occur in the minority of patients with sarcoidosis and is associated with bone marrow infiltration by epithelioid granulomas in about half the cases [56]. Bone marrow involvement can be revealed by FDG PET imaging, which was elegantly demonstrated by De Prost et al. [57]. In their paper, they describe three sarcoidosis patients with symptomatic anemia and increased bone marrow glucose uptake on FDG PET. Bone marrow sarcoidosis was confirmed by bone marrow biopsy. Clinically relevant improvement of anemia that correlated with a significant decrease in bone marrow FDG uptake was observed upon treatment with corticosteroids in two cases.

So, FDG PET/CT may reveal diverse extra pulmonary disease locations in symptomatic sarcoidosis patients, and may aid in diagnosing sarcoidosis as well as in treatment decisions.

## 5. FDG PET/CT in Symptomatic Pulmonary Sarcoidosis

Pulmonary involvement leading to increasing dyspnea, impaired pulmonary function, and substantial radiographic progression is one of the main indications for anti-inflammatory treatment in sarcoidosis [58]. Especially patients with extensive and/or progressive granulomatous inflammation in the lung parenchyma with functional impairment will usually get oral glucocorticoids or an antimetabolite, such as methotrexate, azathioprine, leflunomide, or mycophenolate. For patients who cannot tolerate these drugs or show refractory disease, anti-tumor necrosis factor (TNF) monoclonal antibodies can be an option [59].

Over the past years, special attention has been paid to the utility of FDG PET/CT in the management of pulmonary sarcoidosis and in selecting refractory patients for further anti-inflammatory therapy. Zhao et al. developed a granuloma/hepatoma rat model and were the first to demonstrate significant FDG uptake in the granuloma, which decreased after corticosteroid treatment [60]. Afterwards, the metabolic response to systemic corticosteroids in humans was demonstrated in a small case series [61].

## 6. Pulmonary Function Tests and FDG-Positive Lung Parenchymal Disease in Untreated Patients

In a prospective study of 11 untreated patients with active lung parenchymal disease, FDG PET results were compared with pulmonary function tests [62]. Diffuse active lung parenchymal disease at baseline correlated with a significant decrease in diffusion capacity of the lung for carbon monoxide (DLCO) after six months when untreated. Vital capacity (VC) and forced expiratory volume in one second (FEV1) did not change. Although in a limited number of patients, this finding suggests that inflamed lung parenchyma imaged by FDG PET may affect the pulmonary function. This is supported by the untreated patient group without inflammatory lung parenchyma where neither DLCO, FVC, nor FEV1 changed.

## 7. Pulmonary Function Tests and FDG-Positive Lung Parenchymal Disease in Treated Patients

Keijsers and colleagues were the first to compare pre- and post-therapy FDG PET with lung functional tests [49]. A significant correlation between the decrease in metabolic activity of the lung parenchyma imaged by FDG PET and an improvement of the VC during infliximab treatment was found in 12 patients. This result was confirmed in a prospective analysis of 16 patients with active lung parenchymal disease. In this group, not only FVC increased significantly, FEV1 and DLCO showed a significant improvement as well [62].

A prospective open-label trial in sarcoidosis patients refractory to conventional therapy confirmed that FDG PET is useful in therapeutic decision-making [63]. After 26 weeks of infliximab treatment, mean improvement in FVC was 6.6% predicted (*p* = 0.0007), whereas in the 6 months before treatment, lung function decreased. Maximum standardized uptake values (SUVmax) of the pulmonary parenchyma at baseline correlated significantly with FVC improvement during treatment (*R* = 0.62, *p* = 0.0004), which was confirmed in patients treated with an infliximab biosimilar [64].

A single blind prospective study comparing two doses of corticotropin in chronic pulmonary sarcoidosis recently affirmed a significant fall in SUV of lung lesions in association with improvement of DLCO [65].

These findings support the hypothesis that FDG uptake in the lung parenchyma correlates with clinically relevant, active pulmonary disease and could be predictive for the response to anti-inflammatory treatment.

In fibrotic stages of sarcoidosis, it can be difficult to distinguish between pure end-stage (i.e., extinguished fibrotic stage) and a combined fibrotic-inflammatory stage with ongoing inflammation. Patients with ongoing inflammatory sarcoidosis might benefit from a change or increase of anti-inflammatory therapy. In these patients, FDG PET/CT might be of use in clinical practice. Mostard et al. showed that in patients with pulmonary sarcoidosis and fibrotic changes on HRCT, the majority had PET positive findings, even in the absence of serological inflammation [43]. Out of the 26 patients with fibrotic changes, 22 (85%) had active pulmonary PET findings, of which 18/22 (82%) showed extra thoracic active lesions and 16/22 (73%) showed serological inflammation. In another study, they showed that 73% of sarcoidosis patients with persistent disabling symptoms, had PET-positive findings, even those with radiological stage IV disease. In this group, 66% revealed active sarcoidosis in the lung parenchyma and 80% revealed active extra thoracic lesions [41]. In 20% of the patients, PET was positive without signs of serological inflammatory activity.

## 8. FDG PET/CT in Cardiac Sarcoidosis

Cardiac sarcoidosis can present as a heart block, ventricular arrhythmia, or cardiomyopathy. Given the prognostic and therapeutic relevance of these findings, it is important to identify cardiac involvement. Besides thoracic inflammation, FDG PET/CT is able to detect cardiac involvement as well. A meta-analysis by Youssef et al. [66] yielded an 89% sensitivity and 78% specificity for FDG PET/CT compared to the Japanese Ministry of Health, Labour and Welfare guidelines in the diagnosis of cardiac sarcoidosis.

Although FDG PET/CT is able to detect active granulomas in the myocardium, the physiologic use of glucose by the myocyte complicates image interpretation. In mammals, the myocyte prefers glucose and fatty acids as an energy source. In order to promote the use of fatty acids instead of glucose, it is advised to prepare patients with a high-fat, high-protein, and low-carbohydrate diet followed by a 12-hour fasting state [67,68].

The glucose transporter 4 (GLUT-4) reduces the intracellular glucose uptake. Heparin stimulates the function of the GLUT-4 and therefore reduces the physiologic glucose uptake in the myocyte. Scholtens et al. elegantly demonstrated the additional value of unfractionated heparin when added to the high-fat, high-protein, and low-carbohydrate diet combined with a 12-hour fasting state [69].

However, the use of unfractionated heparin is not without risks. Heparin-induced thrombocytopenia (HIT) is a rare but severe potential side effect, occurring in 0.2%–3.0% of the patients [70]. Therefore, several institutions prefer a prolonged fasting state combined with a high-fat, high-protein, and low-carbohydrate diet. If the additional use of heparin is preferred, a dosage of 50 IE per kilogram bodyweight is recommended, injected 15 minutes prior to the administration of FDG.

The statement paper of the European Association of Nuclear Medicine, the European Association of Cardiovascular Imaging, and the American Society of Nuclear Cardiology provides an overview of the role and correct use of imaging techniques for the evaluation and management of cardiac sarcoidosis [68]. Besides FDG PET/CT for imaging of inflammation, it is recommended to add a myocardial perfusion scan. The myocardial perfusion is reduced in the inflammatory phase as well as in scarred tissue when the inflamed myocardium has turned into fibrosis.

In a prospective study, Blankstein et al. analyzed 118 patients without coronary artery disease who were referred for evaluation of known or suspected cardiac sarcoidosis [71]. FDG PET/CT combined with rubidium-82 myocardial perfusion imaging was performed and adverse events (i.e., death or sustained ventricular tachycardia (VT)) were ascertained. PET was categorized as normal, positive perfusion defect or FDG uptake, and positive perfusion and FDG uptake. They concluded that cardiac PET findings were predictive of adverse events, and the presence of both a perfusion defect and abnormal FDG uptake was associated with a hazard ratio of 3.9. In addition, FDG uptake in the right ventricle was associated with death or VT.

In 23 patients with cardiac sarcoidosis, Osborne et al. correlated the changes in myocardial FDG uptake and left ventricular ejection fraction (LVEF) [72]. Serial FDG PET/CT and rubidium-82 PET were performed to determine the myocardial metabolic activity and LVEF, respectively. They found a significant correlation between the decrease in SUV and improvement of LVEF. In addition, the LVEF increased when the volume of inflamed tissue diminished.

To evaluate the extent and severity of myocardial perfusion and FDG uptake in predicting adverse outcome, Sperry et al. analyzed 203 patients retrospectively [73]. PET was classified as normal perfusion and metabolism, abnormal perfusion or metabolism, or abnormal perfusion and metabolism. Summed rest score in a 17-segment model and FDG uptake expressed as the coefficient of variation (CoV) were used, and associations with death, heart transplant, and ventricular arrhythmia requiring defibrillation were analyzed. Both imaging parameters appeared to be of prognostic importance, and the authors suggested the use of more detailed analysis of FDG PET/CT in the assessment of cardiac sarcoidosis.

## 9. Conclusions

FDG PET/CT can aid in the diagnostic process of sarcoidosis, especially when conventional tests are inconclusive. In addition, FDG PET/CT may reveal treatable active disease, particularly in (fibrotic) pulmonary and cardiac sarcoidosis, and the technique appears to be useful in evaluating treatment efficacy.

## Figures and Tables

**Table 1:** Sensitivity of fluorodeoxyglucose (FDG) positron emission tomography (PET)/CT in assessing sarcoidosis activity.

Reference	Year of Publication	No. of Patients	Study Design	Gold Standard for Active Sarcoidosis	Sensitivity
Yamada et al. [6]	1998	31	Retrospective	Biopsy proven	97%
Nishiyama et al. [10]	2006	18	Retrospective	Biopsy proven	100%
Kaira et al. [11]	2007	24	Prospective	Biopsy proven	100%
Teirstein et al. [5]	2007	137	Retrospective	Biopsy proven	99%
Prager et al. [12]	2008	24	Retrospective	Biopsy proven	96%
Braun et al. [13]	2008	20	Retrospective	Biopsy proven	100%
Keijsers et al. [14]	2009	36	Retrospective	Biopsy proven	94%
Keijsers et al. [15]	2010	77	Retrospective	Biopsy proven	97%
Keijsers et al. [9]	2011	34	Prospective	Biopsy proven	97%
Maturu et al. [16]	2014	88	Prospective	Biopsy proven	99%
Guleria et al. [17]	2014	25	Prospective	Biopsy proven	96%
Simonen et al. [7]	2015	57	Retrospective	Biopsy proven	89%
Norikane et al. [8]	2017	20	Retrospective	Clinical and/or histologically proven	100%
